# Neodymium-doped mesoporous silica nanoparticles promote bone regeneration via autophagy-mediated macrophage immunomodulation

**DOI:** 10.1016/j.mtbio.2025.102198

**Published:** 2025-08-12

**Authors:** Qing Zhang, Duraipandy Natarajan, Weijian Gao, Haokun He, Shuguang Cheng, Yin Xiao, Marco N. Helder, Sujuan Zeng, Richard T. Jaspers, Janak Lal Pathak

**Affiliations:** a School and Hospital of Stomatology, Guangdong Engineering Research Center of Oral Restoration and Reconstruction, Guangzhou Key Laboratory of Basic and Applied Research of Oral Regenerative Medicine, Guangzhou Medical University, Guangzhou, 510182, China; b Laboratory for Myology, Department of Human Movement Sciences, Faculty of Behavioural and Movement Sciences, Vrije Universiteit Amsterdam, Amsterdam Movement Sciences, 1081 BT, Amsterdam, the Netherlands; c School of Biomedical Engineering, Guangzhou Medical University, Guangzhou, 511436, Guangdong, China; d School of Medicine and Dentistry & Institute for Biomedicine and Glycomics, Griffith University, Gold Coast, QLD, 4222, Australia; e Department of Oral and Maxillofacial Surgery/Oral Pathology, Amsterdam University Medical Centers and Academic Centre for Dentistry Amsterdam (ACTA), Vrije Universiteit Amsterdam, Amsterdam Movement Sciences, 1081 HV, Amsterdam, the Netherlands

**Keywords:** Neodymium doped mesoporous silica nanoparticles, Immunomodulation, Autophagy, Angiogenesis, Osteogenesis, Osteolysis

## Abstract

Rare earth nanomaterials, especially those incorporating neodymium, hold great potential for bone regeneration, but their clinical application is limited by insufficient understanding of immunomodulatory effects and potential toxicity concerns. To address this, we developed neodymium-doped mesoporous silica nanoparticles (NDMSN) to modulate macrophage autophagy and polarization. NDMSN exhibited uniform dispersion with an average size of 103 nm. NDMSN displayed low cytotoxicity in M0 macrophages and effectively suppressed pro-inflammatory responses in M1 macrophages. This was evidenced by the inhibition of pro-inflammatory markers (IL-6, IL-1β, and iNOS) and the promotion of anti-inflammatory markers (IL-4, IL-10, and CD206). Autophagy activation was confirmed by upregulated expression of P62, LC3A, BECLIN1, and ATG7, and the anti-inflammatory effects were attenuated upon autophagy inhibition with 3-methyladenine, highlighting autophagy's essential role. Conditioned medium from NDMSN-treated M1 macrophages exhibited pro-angiogenic activity in human umbilical vein endothelial cells by enhancing tube formation and elevating angiogenic gene expression, while showing pro-osteogenic potential in mouse bone marrow mesenchymal stromal cells. In vivo, NDMSN mitigated LPS-induced bone destruction in a mouse calvarial osteolysis model and suppressed osteoclast differentiation. Its osteogenic capacity was further validated in a zebrafish calvarial defect model. These findings demonstrate that NDMSN is a promising immunomodulatory and osteogenic nanomaterial, offering a novel therapeutic strategy for bone regeneration.

## Highlights

• Neodymium-doped mesoporous silica nanoparticles (NDMSN) showed biocompatibility and macrophage immunomodulatory properties.
• NDMSN-induced anti-inflammatory response in macrophages was attributed to autophagy.
• Conditioned medium from NDMSN-treated macrophages promoted angiogenic and osteogenic differentiation of precursor cells.
• In a murine calvarial osteolysis model, NDMSN effectively reversed LPS-induced bone erosion.
• Zebrafish cranial defect model confirmed the bone regenerative potential of NDMSN.

## Graphical abstract

**Figure:**
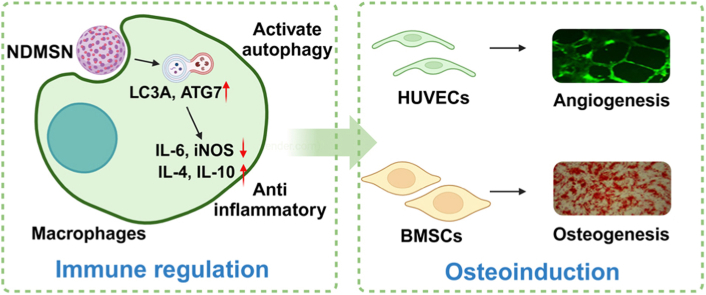

## 1 Introduction

Severe bone defects resulting from trauma, infection, or congenital disorders present significant clinical challenges, as bone regeneration involves a complex interplay of biological processes [1]. Ideal therapeutic materials must not only provide structural support but also actively orchestrate the cellular and molecular events driving healing [2,3]. Rare earth elements (REEs) have emerged as promising candidates due to their unique physicochemical properties, including osteoconductivity, osteoinductivity, and pro-angiogenic effects, attributed to their large ionic radii and electronic configurations [4,5]. Their similarity to Ca^2+^ enables strong bone affinity, while their redox activity supports applications in bioimaging, drug delivery, and antioxidant therapy [5,6]. For instance, cerium oxide nanoparticles mitigate oxidative stress and stimulate osteogenesis, underscoring their therapeutic versatility [7]. However, current research has predominantly focused on the direct osteogenic effects of REEs, such as mineral deposition and osteoblast activation [8], neglecting their osteoimmunomodulatory potential, a critical factor in bone repair. Macrophages, key regulators of immunomodulation during bone regeneration, significantly influence the bone defect healing microenvironment [9,10]. Yet, the interactions between rare earth nanomaterials and macrophages remain poorly understood, limiting their clinical translation.

To bridge this gap, we investigate the immunomodulatory potential of rare earth nanomaterials, focusing on macrophage polarization during bone defect healing. Macrophages dynamically shift between pro-inflammatory (M1) and anti-inflammatory (M2) phenotypes, critically shaping the osteoimmune microenvironment. While excessive M1 activity exacerbates bone resorption, controlled M1 responses or a transition to the M2 phenotype promote repair by stimulating osteogenesis, angiogenesis, and tissue remodeling [11,12]. Central to this balance is autophagy, a lysosomal degradation pathway that regulates macrophage polarization and inflammatory signaling [[13], [14], [15]]. Emerging evidence suggests that autophagy induction can skew macrophages toward a pro-regenerative phenotype, offering a promising lever to optimize bone regeneration [[16], [17], [18]]. By targeting this mechanism, rare earth nanomaterials could unlock novel strategies to harness the macrophage-mediated immunomodulation for enhanced bone repair.

Targeting macrophage behavior represents a powerful approach to orchestrate bone regeneration, highlighting the need to elucidate how rare-earth nanomaterials modulate these immune cells. Among lanthanides, neodymium (Nd) stands out for its autophagy-inducing properties [19] and demonstrated efficacy in tissue engineering applications [[20], [21], [22]]. Nd-doped bioactive glass, for instance, accelerates wound healing [22]. While Nd nanoparticles promote redox-mediated angiogenesis in endothelial cells through shape-dependent mechanisms [23]. Notably, Nd has also been shown to trigger autophagy in cancer cells [19], suggesting its broader regulatory potential. However, its role in osteoimmunomodulation, particularly in macrophage polarization, remains largely unknown, creating a critical knowledge gap.

Despite these promising characteristics, clinical translation of Nd-based nanomaterials faces significant challenges. High-dose Nd oxide formulations exhibit substantial cytotoxicity, inducing oxidative stress and cell death [[24], [25], [26]]. These adverse effects underscore the urgent need for innovative material designs that preserve Nd's therapeutic benefits while minimizing its toxicity, which is a key focus of our study. Doping mesoporous silica nanoparticles (MSNs) with Nd presents an innovative strategy to overcome the cytotoxicity limitations of rare earth materials [27]. MSNs offer distinct advantages for biomedical applications, including an exceptionally high surface area, tunable pore architecture, and proven biocompatibility characteristics that make them ideal platforms for drug delivery and bioactive element incorporation [[28], [29], [30]]. By structurally integrating Nd into the MSN matrix, we can simultaneously mitigate its cytotoxic effects while preserving its beneficial osteoimmunomodulatory properties. This engineered approach creates an optimal balance between biological safety and therapeutic efficacy, enabling the full exploitation of Nd's bone regenerative potential.

In this study, we developed Nd-doped mesoporous silica nanoparticles (NDMSN) as a multifunctional bone regeneration platform, focusing on their immunomodulatory and osteogenic properties. We systematically investigated: (1) the autophagy-mediated anti-inflammatory effects of NDMSN on M1 macrophage polarization in vitro; (2) their downstream influence on osteogenic differentiation and angiogenic processes through macrophage crosstalk; and (3) their therapeutic efficacy *in vivo* using both mouse calvarial osteolysis and zebrafish bone defect models. Our comprehensive approach bridges the gap between material design and immunomodulation, offering new insights into rare earth nanomaterial applications for bone tissue engineering.

## 2 Materials and methods

### 2.1 Materials

Tetraethyl orthosilicate (TEOS), aqueous ammonia (NH_3_H_2_O), anhydrous ethanol, cetyltrimethylammonium bromide (CTAB), Nd nitrate, anhydrous ethanol, dexamethasone, pentobarbital sodium, β-glycerophosphate, 4ʹ,6-dimidazole-2-phenylindole (DAPI), and lipopolysaccharide (LPS) were bought from Sigma Aldrich company (USA). Fluorescein isothiocyanate (FITC) and 3-Aminopropyltriethoxysilane (APTES) were purchased from Aladin (China). Agar powder was purchased from Huankai Microbial Technology Co., Ltd. (Guangzhou, China). Tricaine (MS-222) was obtained from Sigma (USA). Hydrogels were obtained from Yuanman Biotechnology Co., Ltd. (Guangzhou, China). The 3-methyladenine (3-MA) was purchased from Selleck (s2767, USA). Rabbit anti-osteopontin antibody (OPN) (ab75285, Abcam, UK), Rabbit anti-Mannose receptor antibody (CD206) (ab64693, Abcam, UK), rabbit anti-iNOS antibody (ab178945, Abcam, UK), CD86 monoclonal Antibody (17-0862-82, Thermo Fisher Scientific, USA), rabbit anti-p62 antibody (ab207305, Abcam, UK), rabbit anti-LC3B antibody (ab192890, Abcam, UK), rabbit anti-Beclin-1 antibody (ab210498, Abcam, UK), CD31 antibody (GB113151, Servare Biotech Inc, China) and secondary antibody anti-rabbit IgG (1:5000, Abcam) were purchased. The medical hydrogel was obtained from Guangdong Technology Development Co., Ltd. (China).

### 2.2 Synthesis and characterization of NDMSN

NDMSN were synthesized using the sol-gel method [31]. Briefly, CTAB was dissolved in water, followed by adding TEOS, stirring, and then adding ammonia as a catalyst. After 24 h, the mixture was centrifuged at 5000g for 10 min, and the pelleted solid material was added to a solution of absolute ethanol with Nd nitrate at a concentration of 100 mM. The container was sealed and stirred for 3 days, whereafter stirring was continued, and the container was opened until the ethanol was evaporated entirely. The resulting powder was transformed into a muffle furnace for calcination at 600 °C for 5 h to obtain the final product NDMSN. Transmission electron microscopy (TEM) and energy dispersive spectrometry (EDS) were used to detect nanoparticle element distribution and content. Dynamic Light Scattering (DLS) was used to analyze the size and zeta potential of NDMSN.

### 2.3 Detection of the release ratio of Nd ions from NDMSN

The NDMSN were dispersed in DMEM medium (37 °C) at two concentrations (25 and 100 μg/mL) to establish dynamic release profiles. Following scheduled sampling intervals (1, 3, 5, 7, and 14 d), aliquots underwent centrifugation with 50 % medium replacement using fresh DMEM. Quantitative analysis of Nd ion liberation was performed via ICP-MS, with cumulative release percentages calculated relative to the initial Nd content in NDMSN.

### 2.4 Cell culture and maintenance

Human umbilical vein endothelial cells (HUVECs) and the murine macrophage cell line (RAW264.7) were acquired from the National Collection of Authenticated Cell Cultures (Shanghai, China). In an incubator with 5 % CO2 and 95 % air environment at 100 % humidity, cells were cultivated and grown in high-glucose DMEM (Gibco, USA) medium supplemented with 10 % fetal bovine serum (Gibco, USA) and 1 % Penicillin-Streptomycin solution (Gibco, USA) at 37 °C. BMSCs from mice were isolated in the manner previously reported [32]. In short, high glucose DMEM medium supplemented with 20 % fetal bovine serum and 1 % Penicillin-Streptomycin solution was used to enable the BMSCs to adhere to tissue culture-treated surfaces, upon which the floating hematopoietic cells were rinsed away. Cells were grown in an incubator under the same conditions as described above. Following 90 % confluency, 0.25 % trypsin was used to passage the cells.

### 2.5 Cell viability and phagocytosis detection of NDMSN in macrophages

By cultivating roughly 2 × 10^4^ macrophage (RAW 264.7) cells/well in 96-well plates with 0, 25, 50, 100, 200, and 400 μg/mL of NDMSN for 24, 48, and 72 h, the impact of increasing NDMSN dosages on cell viability was examined. Cell viability was assessed using the cell counting kit-8 assay (CCK8, BestBio, China) as previously described [33].

FITC labeling of NDMSN was executed according to our previous report [33]. Briefly, FITC and APTES were mixed with anhydrous ethanol and swirled in the dark to produce APTES-FITC. Subsequently, 20 μg of NDMSN were added and mixed for 2 h without light. After centrifugation at 5000*g* for 10 min, PBS was used to rinse the FITC-labeled NDMSN.

Cell plates containing cells seeded at a density of 2 × 10^4^ cells/well were used to measure the absorption of NDMSN-FITC particles by macrophages. The culture solution was replaced with one that included NDMSN-FITC particles at a concentration of 25 μg/mL (chosen based on the cell viability assay) after 6 h of incubation. The plates were then gently shaken for 1 h at 37 °C. Following a further 6 h of incubation, the cells were rinsed three times with PBS before the nuclei were stained for 5 min with DAPI (Blue). A Leica DM8 fluorescence microscope and Flow cytometry were used to capture the images and FITC-positive cells.

### 2.6 RT-qPCR analysis

The immunomodulatory effects of mesoporous silica nanoparticles (MSNs) were evaluated through comprehensive gene expression analysis in macrophages, human umbilical vein endothelial cells (HUVECs), and bone marrow stromal cells (BMSCs). Macrophages were treated with different groups: (1) Control (PBS), (2) LPS (100 ng/mL LPS for 24 h), (3) LPS + LNDMSN (LPS followed with LNDMSN: high dose of NDMSN (25 μg/mL)), and (4) LPS + HNDMSN (LPS followed with HNDMSN: high dose of NDMSN (100 μg/mL). To investigate the immunomodulation effect of MSN, the MSN (25 μg/mL) was used as a control group. To investigate the autophagy-induced immunregulation, 3-MA (5 mM) was used to inhibit autophagy in M1 macrophages 1 h before NDMSN treatment. HUVECs and BMSCs were treated with condition medium derived from different groups of treated macrophages. After treating macrophages with MSN or NDMSN for 24 h, the culture medium was replaced with serum-free medium without nanoparticles. Following an additional 12 h incubation, the culture supernatant was collected and centrifuged to remove residual nanoparticles and cellular debris. **For the conditioned angiogenic differentiation culture medium:** the macrophage culture supernatant was then mixed 1:1 with fresh ECM (10 % fetal bovine serum, 1 % endothelial cell growth supplement (ECGS), and 1 % penicillin/streptomycin solution) and used for HUVECs culture. **For the conditioned osteogenic differentiation culture medium:** the macrophage culture supernatant was mixed with a 1:1 mixture of osteogenic induction medium (2 × OM: DMEM medium with 20 % fetal bovine serum, 1 % penicillin/streptomycin solution, 20 nM dexamethasone, 100 μg/mL ascorbic acid, and 20 mM β-sodium glycerophosphate) and used for BMSCs culture. Four types of media were prepared for subsequent experiments: (a) Control: non-conditioned medium; (b) M-CM: PBS-treated group; (c) ML-CM: LPS-treated group; (d) MLN-CM: LPS + NDMSN-treated group. Conditioned medium from MSN treatment on LPS-induced macrophages (MLM-CM) was also collected and used for osteogenic differentiation of BMSCs. Total RNA was extracted from BMSCs, HUVECs, and macrophages using an RNA extraction column kit (YEASEN, China). A cDNA synthesis kit (YEASEN, China) was then used to create complementary DNA (cDNA) from 1 μg of total RNA. A SYBR Green Master mix (Takara, Japan) was used for quantitative RT-PCR on a LightCycler 480 (Roche, USA). Every experiment was triple-replicated and adhered to MIQE guidelines. The expression of target genes was normalized by using GAPDH as an internal reference. The normalized control group's values were contrasted with those of the experimental groups. Table 1 lists mouse-specific primers, which were used for mouse-derived macrophages RAW264.7 cells and BMSCs, and Table 2 lists human-specific primers for HUVECs. The detailed primer sequences used for RT-qPCR are as follows.

**Table 1:** Mouse original primer sequences used in the study.

Gene	Forward Primer (5' - 3′)	Reverse Primer (5′- 3′)
IL-6	AGCCCACCAAGAACGATAG	GGTTGTCACCAGCATCAGT
IL-1β	TGAGGACATGAGCACCTTC	GGGAACGTCACACACCA
iNOS	GCCCAGGAGGAGAGAGAT	GCAAAGAGGACTGTGGCT
IL-4	CCCCCAGCTAGTTGTCATCC	AGGACGTTTGGCACATCCAT
IL-10	GCCCTTTGCTATGGTGTC	TCTCCCTGGTTTCTCTTCC
CD206	CCCTGCTACTGAACCTCCT	AGCCTGACCCCAACTTCT
ATG7	ACCTCGCTGGGACTTGTGC	GGTGAATCCTTCTCGCTCGT
LC3	CCTGTCCTGGATAAGACCAAGTT	CTCCTGTTCATAGATGTCAGCGAT
BECLIN1	CAGTACCAGCGGGAGTATAGTGA	TGTGGAAGGTGGCATTGAAGA
LAMP2	CTACAGCCCAGGAGTGTTCG	CCTGAAAGACCAGCACCAAC
CD31	AACAGTGTTGACATGAAGAGCC	TGTAAAACAGCACGTCATCCTT
EMN	ATCCGGGCACACCAGAAAAT	CTTTTCCACGCTTGGTGCAT
VEGF	AGGGCAGAATCATCACGAAGT	AGGGTCTCGATTGGATGGCA
VEGFR2	GGCCCAATAATCAGAGTGGCA	CCAGTGTCATTTCCGATCACTTT
COL-l	ATGCCGCGACCTCAAGATG	TGAGGCACAGACGGCTGAGTA
OPN	ACCATGCAGAGAGCGAGGATT	GGGACATCGACTGTAGGGACG
OCN	AGCAGCTTGGCCCAGACCTA	TAGCGCCGGAGTCTGTTCACTAC
GAPDH	TGTTTCCTCGTCCCGTAGA	ATCTCCACTTTGCCACTGC

**Table 2:** Human original primer sequences used in the study.

Gene	Forward Primer (5' - 3′)	Reverse Primer (5′- 3′)
CD31	AACAGTGTTGACATGAAGAGCC	TGTAAAACAGCACGTCATCCTT
EMN	ATCCGGGCACACCAGAAAAT	CTTTTCCACGCTTGGTGCAT
VEGF	AGGGCAGAATCATCACGAAGT	AGGGTCTCGATTGGATGGCA
VEGFR2	GGCCCAATAATCAGAGTGGCA	CCAGTGTCATTTCCGATCACTTT
COL-l	ATGCCGCGACCTCAAGATG	TGAGGCACAGACGGCTGAGTA
OPN	ACCATGCAGAGAGCGAGGATT	GGGACATCGACTGTAGGGACG
OCN	AGCAGCTTGGCCCAGACCTA	TAGCGCCGGAGTCTGTTCACTAC
GAPDH	GCACCGTCAAGGCTGAGAAC	TGGTGAAGACGCCAGTGGA

### 2.7 Immunofluorescence

Immunofluorescence staining researches were conducted to test pro-inflammatory (M1 marker, iNOS), anti-inflammatory (M2 marker, CD206), and blood vessel marker (CD31) in macrophages. Macrophages were stimulated with 100 ng/mL LPS for 24 h to induce M1 polarization, followed by treatment with 25 μg/mL NDMSN for 8 h. This treatment regimen was selected to allow sufficient time for both polarization establishment and nanoparticle-mediated effects while maintaining cell viability. They were washed three times then fixed with 4 % paraformaldehyde, permeabilized with Triton-X100 (0.2 %), and non-specific protein binding blocked with BSA-PBS (3 %). After that, primary antibodies were used to incubate cells, then washed before secondary antibodies conjugated with Cy3 and FITC. After washing, cells were co-stained by 1 μg/mL of DAPI, visualized by confocal microscopy, and analyzed using Leica software.

### 2.8 Flow cytometry test

The adherent macrophages were washed by gentle pipetting to remove non-adherent cells, then centrifuged to collect the cells. The obtained cells were subsequently washed once with DMEM (Gibco, USA) before proceeding to antibody staining. For surface marker detection, cells were stained with the following fluorescently conjugated antibodies: anti-CD86 monoclonal antibody (clone GL1), APC-Cy7-labeled secondary antibody (Thermo Fisher Scientific, USA), and rabbit anti-mannose receptor antibody (CD206. ab64693, Abcam, UK) conjugated with Alexa Fluor 647 (APC). Following a 30 min incubation in the dark, the stained macrophages underwent two additional washes with DMEM to remove unbound antibodies. Cellular fluorescence was then statistically analyzed using a BD FACSVerse flow cytometer (BD Biosciences, San Jose, CA) equipped with three laser lines (488 nm, 640 nm, and 405 nm). All flow cytometry data were processed and visualized using FlowJo (v. 10.8.1, TreeStar, Ashland, OR), with compensation matrices applied to correct for spectral overlap between fluorophores.

### 2.9 Biological transmission electron microscopy (TEM)

The uptake of NDMSN in macrophage cells and the development of autophagosomes were observed by TEM. After being cultivated with 100 ng/mL LPS for 24 h to promote inflammation, macrophages were cultured with and without NDMSN for 6 h. Following an overnight fixation with pre-cooled glutaraldehyde, the cells were then prepared for TEM sample. As previously mentioned, TEM (Tecnai Spirit, FEI, Hillsboro, USA) was used to capture the pictures at highest resolution and magnification [33].

### 2.10 Enzyme-linked immunosorbent assay (ELISA)

Macrophages were grown in serum-free media under three conditions (PBS, LPS, and LPS + LNDMSN) for 6 h. The conditioned media were collected and used for cytokine expression analysis by ELISA. The manufacturer's instructions were followed to measure TGF-β and IL-10 levels with an ELISA kit (Cloud-Clone Corp., Wuhan, China). The TGF-β and IL-10 concentration was calculated by a standard curve and represented as pg/mL supernatant.

### 2.11 Werstern blot (WB)

M1 macrophages (LPS-treated) were cultured in DMEM medium containing PBS or NDMSN for 12 h. The cells were lysed with RIPA buffer (containing protease inhibitors, PMSF), and the proteins were collected for Western blot analysis. The total protein concentration was determined using a BCA assay kit (Beyotime, China). The WB experiment was then performed following previously described methods. 20 μg of protein was separated by SDS-PAGE (10 %) and transferred onto a PVDF membrane (Millipore) via wet transfer in buffer. The membrane was blocked with 5 % skim milk for 1 h, washed, and then incubated overnight with primary antibodies (p62, LC3B, and Beclin-1) in a diluted solution. After incubation, the membrane was washed three times with PBST and further incubated with horseradish peroxidase (HRP)-conjugated secondary antibodies for 2 h. Finally, protein bands were visualized using ECL chemiluminescence, and images were captured. GAPDH served as the loading control, and band intensity was quantified using ImageJ software.

### 2.12 Tube formation assay

The tube formation assay was performed to evaluate the effects of NDMSN-treated macrophages on angiogenesis. Prior to the experiment, 50 μL of Matrigel (0.2 mg/mL) was evenly coated onto μ-Slide Angiogenesis plates and polymerized at 37 °C under 5 % CO_2_ for 30 min. Simultaneously, HUVECs were trypsinized and seeded in 24-well plates at a density of 2 × 10^4^ cells/cm^2^. The HUVECs were treated with conditioned angiogenic differentiation medium as mentioned in Methods 2.6. After overnight incubation at 37 °C with 5 % CO_2_, cells were stained with 2 μg/mL Calcein AM, followed by PBS washing to remove excess dye. Tube formation was visualized and imaged using fluorescence microscopy, with quantitative analysis of node number, segment length, mesh count, and total mesh area performed using Angioquant software.

### 2.13 Migration assay

A modified scratch assay was employed to evaluate the effects of different treatments on endothelial cell migration. Briefly, HUVECs were digested with 0.25 % trypsin-EDTA, resuspended in DMEM supplemented with 10 % fetal bovine serum, and seeded at a density of 2 × 10^5^ cells per well in 24-well plates pre-coated with 0.1 % gelatin. Cells were cultured at 37 °C in a humidified incubator with 5 % CO_2_ until confluence was reached. A linear scratch was created using a sterile 200 μL pipette tip, and detached cells were gently removed with PBS (pH 7.4). Cells were then treated with one of the following: PBS (control), M-CM, ML-CM, and MLN-CM. All conditioned media were mixed 1:1 with serum-free DMEM and supplemented with 1 % penicillin-streptomycin. Images were acquired at 0 and 12 h using an inverted phase-contrast microscope (Leica DMi8), and cell migration area and scratch closure rate were quantified using ImageJ software. Each group was performed in triplicate, and three random fields were imaged per well. All procedures were standardized to ensure data consistency.

### 2.14 Osteogenic differentiation of bone marrow-derived mesenchymal stem cells

Mouse bone marrow-derived mesenchymal stem cells (BMSCs) were seeded at a density of 2 × 10^4^ cells/cm^2^ into either 6-well or 48-well culture plates, depending on subsequent experimental requirements. After allowing cells to adhere overnight in standard growth medium, the cells were treated with conditioned osteogenic differentiation medium as mentioned in Methods 2.6. Cultures were maintained in a humidified incubator at 37 °C with 5 % CO_2_. The mixed medium was refreshed every two days to maintain osteogenic stimuli and nutrient supply. At predetermined time points, day 4 and day 7 of differentiation, cells were harvested for downstream analyses, including gene expression, staining, or protein quantification, depending on the experimental design.

### 2.15 Alkaline phosphatase (ALP) and mineralized matrix

BMSCs were subjected to alkaline phosphatase (ALP) staining after 10 days of conditioned medium treatment. Prior to staining, cells were gently washed twice with PBS to remove residual culture medium, followed by fixation with 4 % paraformaldehyde for 10 min. The color development was then performed using BCIP/NBT substrate kit (Nanjing, China) according to the manufacturer's protocol. After color reaction completion, the samples were rinsed with distilled water and photographed for documentation. ARS staining (Sigma) was performed to visualize and semi-quantitatively assess the mineralized matrix in the cultured BMSCs. ALP and mineralized matrix were quantified as described previously [34].

### 2.16 LPS-induced mouse calvarial osteolytic model

The animal experiment involving NDMSN intervention for bone regeneration was approved by the Animal Ethics Committee of Guangdong Huawei Testing Co., Ltd. (Approval No. 20210603). Based on established methods in the literature [35,36], we developed an LPS-induced mouse calvarial bone resorption model. A total of 24 six-week-old male C57BL/6 mice were randomly devided into four groups: (a) Control group, receiving 100 μL of PBS via subcutaneous injection; (b) LPS group, receiving 100 μL of 10 mg/kg LPS; (c) LNDMSN group, receiving 100 μL of a mixture containing 10 mg/kg LPS and 25 μg/kg NDMSN; (d) HNDMSN group, receiving 100 μL of a mixture containing 10 mg/kg LPS and 100 μg/kg NDMSN. Throughout the experiment, no adverse reactions or mortality were observed in any group. The mice were mildly anesthetized, and subcutaneous injections were administered every 3 days in the calvarial region at the midline between the ears and eyes for a total duration of 10 days. Upon completion of the experiment, the mice were euthanized by cervical dislocation, and calvarial samples were collected and fixed in 4 % paraformaldehyde for 48 h. Subsequently, the calvarial specimens were scanned using a micro-CT scanner (Skyscan 1076, Bruker; resolution 10 μm; X-ray settings: 60 kV/100 μA; exposure time 300 ms). Three-dimensional reconstructions of the skull were obtained using the provided software, and a 4 mm^2^ region of interest in the bone resorption area was selected for analysis. Bone morphology parameters, including bone volume fraction (BV/TV), bone surface-to-volume ratio (BS/TV), bone mineral density (BMD), and trabecular separation (Tb.Sp), were assessed using the CT analysis system (CTAn v1.11.10.0, SkyScan). For histological analysis, calvarial tissues were fixed in 4 % paraformaldehyde, decalcified in 10 % EDTA solution for two weeks, and subsequently embedded in paraffin and sectioned to a thickness of 4 μm. Hematoxylin and eosin (H&E) staining was performed to evaluate bone defect repair, and tartrate-resistant acid phosphatase (TRAP) staining was used to quantify osteoclasts [37]. Image analysis was performed using ImageJ software.

### 2.17 Zebrafish skull defect model

The zebrafish were reared under standardized conditions, including a temperature of 28 ± 0.5 °C, a photoperiod of 14 h light and 10 h dark, and a diet consisting of live brine shrimp and flake food provided twice per day. Zebrafish were randomly divided into three groups, including the control group (Hydrogel, n = 10), LNDMSN (low dose of NDMSN, NDMSN: hydrogel = 1:1, n = 10), and HNDMSN (high dose of NDMSN, NDMSN: hydrogel = 2:1, n = 10). Zebrafish were first anesthetized with 0.05 % tricaine methane sulfonate, after which a stereotactic craniotomy system was employed to generate a standardized circular cranial defect (∼200 μm in diameter). Post-surgery, the fish were transferred to water for a 10-min recovery period with unrestricted movement. Then, anesthetized again, the defect was made, and immediately coated with hydrogel or nanoparticle-containing hydrogel. To evaluate bone regeneration, zebrafish anesthetized in 0.05 % tricaine methane sulfonate solution were wrapped and fixed using Agar to keep them in a swimming posture. The zebrafish were imaged with an Optical coherence tomography (OCT) imaging system by adjusting the appropriate position to obtain OCT cross-sectional images. OCT was performed at 0, 3,6,9,12,15,18, and 21 days post-surgery. Immediately after each OCT imaging, all zebrafish were returned to the original culture conditions. In addition, on days 0 and 21, the zebrafish were subjected to 3D imaging under anesthesia, as described above, to ensure good 3D image quality and evaluate the recovery of the zebrafish skull injury.

### 2.18 Statistical analysis

All statistical analyses were performed using GraphPad Prism software (version 8.0). Experimental data, collected from a minimum of three biological replicates, are reported as means ± standard deviation. Prior to analysis, data distribution normality was confirmed through Shapiro-Wilk testing. Parametric data were subjected to one-way ANOVA with two-tailed testing (α = 0.05), followed by either Bonferroni's multiple comparisons test or Dunnett's test when appropriate. The interaction between temporal factors and treatment conditions in bone defect diameter measurements was assessed using two-way ANOVA. For non-parametric data, the Mann-Whitney *U* test was applied for comparisons between two groups, while the Kruskal-Wallis test followed by Dunn's post hoc test was used for comparisons among multiple groups. Statistical significance was defined at p < 0.05 for all tests.

## 3 Results

### 3.1 NDMSN characterization, cell viability, and endocytosis

**Fig. 1:**
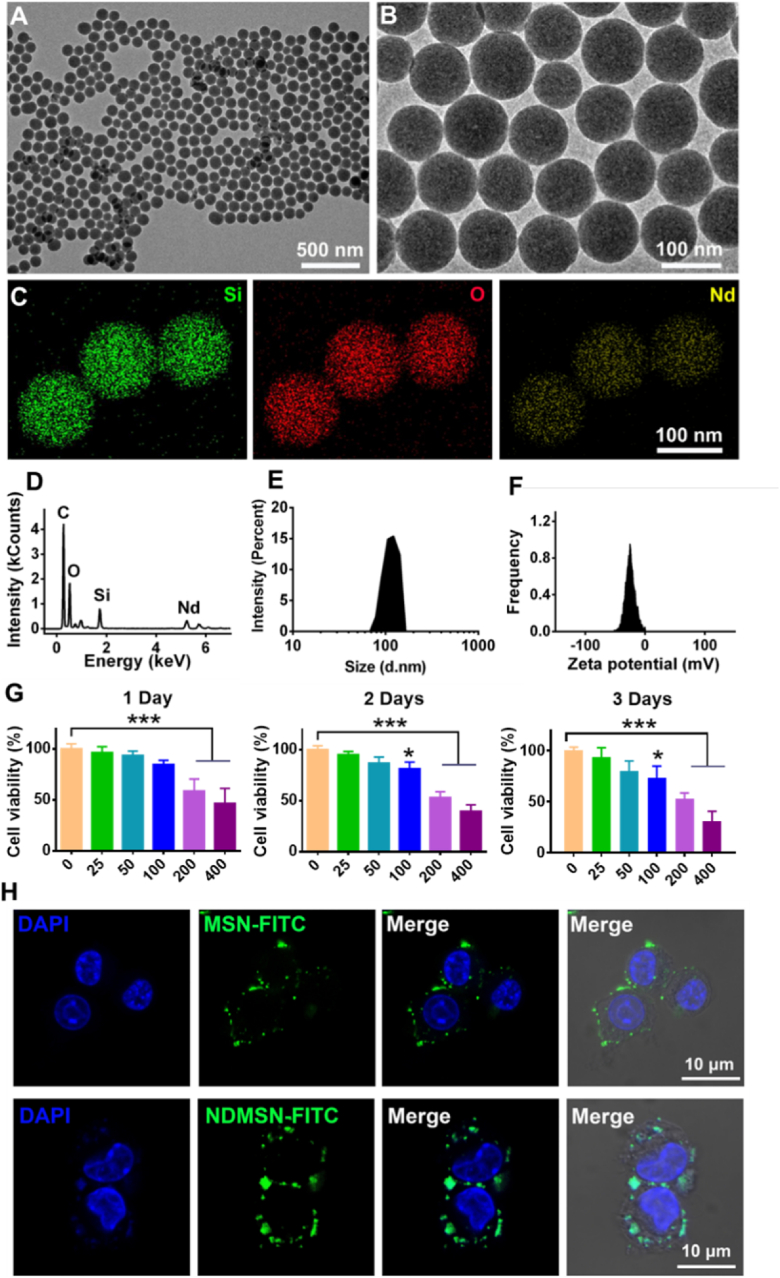
**Physicochemical characterization and biocompatibility evaluation of neodymium-doped mesoporous silica nanoparticles (NDMSN).** (A, B) TEM images of NDMSN at low and high magnification. (C, D) EDS mapping and analysis. Si (Red), O (Green), and Nd (Yellow). (E) The particle size and (F) zeta potential of NDMSN. (G) The cell viability of NDMSN-treated macrophages was time and concentration-dependent. (H) Macrophages phagocytosed MSN-FITC and NDMSN-FITC after 6 h. Data represent mean ± SD (n = 3); Significant difference between the groups, ∗p < 0.05 and ∗∗∗p < 0.001.

The TEM images of the nanoparticles showed that NDMSN was successfully synthesized with good dispersity (Fig. 1A). The high magnification image showed that nanoparticles with a reasonably homogeneous particle size of around 100 nm in diameter (Fig. 1B). Elemental analysis confirmed that the silicon (Si), oxygen (O), and Nd were well dispersed into the nanoparticles (Fig. 1C). According to the elemental analysis data, the Nd content was 0.9 % (Fig. 1D). The hydrodynamic size of the NDMSN was around 105 nm with 0.104 polydispersity index (PDI) tested by dynamic light scattering (DLS) (Fig. 1E). The zeta-potential was about −24 mV as tested by a particle-size potentiometer (Malvern, UK) in deionized water (Fig. 1F), The Nd ion release from NDMSN was tested; the ion release profile shows a consistent release of Nd ions from NDMSN in DMEM solution over 14 days, reaching a cumulative release of 33 % (Fig. S1).

A CCK-8 tested the biocompatibility of NDMSN in macrophages. Different NDMSN concentrations were tested (Fig. 1G). No significant toxicity was observed up to 100 μg till day 2. However, macrophage viability significantly decreased after treatment at 100 μg for 3 days, indicating the dose-dependent and time-dependent cytotoxic effect of NDMSN on macrophages (Fig. 1G). At concentrations below 100 μg/mL, macrophage viability remained consistently above 90 %, compared to the control group showing no obvious difference. However, at 100 μg/mL, the viability decreased to 80.2 % after 2 days and declined to 72.6 % after 3 days of treatment (Fig. 1G). Based on the observed impacts of NDMSN on cell viability, we selected two concentrations for further testing: LNDMSN at 25 μg/mL and HNDMSN at 100 μg/mL. Fluorescence images showed that macrophages phagocytosed the NDMSN after 6 h, and both MSN and NDMSN nanoparticles were dispersed in the cytoplasm surrounding the cell nucleus (Fig. 1H). Moreover, flow cytometry was also used to verify the nanoparticles’ uptake by macrophages. The results indicated that around 95 % macrophages phagocytosed both MSN and NDMSN (Fig. S2).

### 3.2 NDMSN switched macrophages from M1 to M2 phenotype

**Fig. 2:**
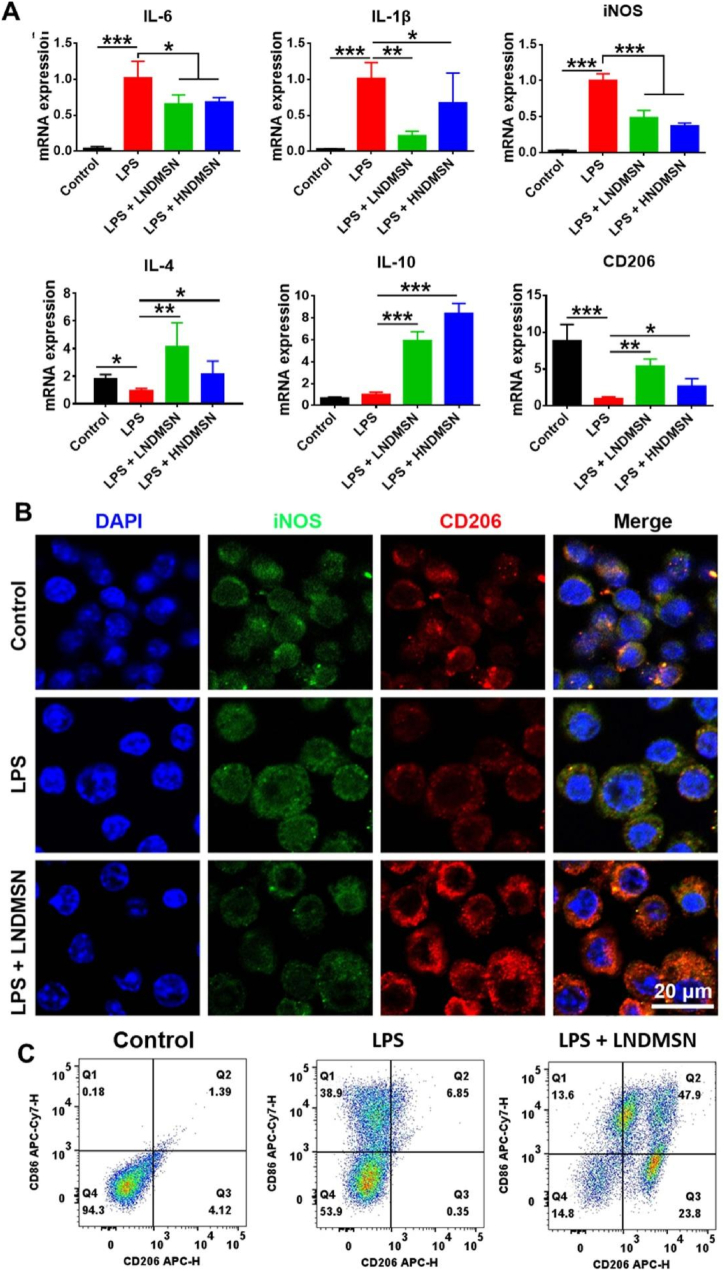
**NDMSN switches M1 to M2 macrophage polarization.** (A) RT-qPCR results of proinflammatory genes (IL-6, IL-1β, and iNOS) and anti-inflammatory genes (IL-4, IL-10, and CD206). (B) Immunofluorescence staining analysis of iNOS (green) and CD206 (red) expression in macrophages. (C) The flow cytometry was used to test the CD86 and CD206 positive cells in macrophages treated with PBS, LPS, and LPS + LNDMSN, respectively. Data are mean ± SD, n = 3. Significant difference between the groups, ∗p < 0.05, ∗∗p < 0.01, and ∗∗∗p < 0.001.

LPS was used to induce M1 macrophage polarization. The quantitative RT-qPCR analysis showed significant anti-inflammatory effect. Both LNDMSN and HNDMSN treatments significantly downregulated pro-inflammatory genes (IL-6, IL-1β, and iNOS), but for IL-1β, the downregulation was most pronounced in the LNDMSN group (Fig. 2A). Notably, LNDMSN- and HNDMSN-treated M1 macrophages displayed marked upregulated expression levels of the anti-inflammatory genes (IL-4, IL-10, and CD206) compared with the untreated group (Fig. 2A). It is worth noting that MSN alone did not exhibit anti-inflammatory effect in M1 macrophages, but the NDMSN could improve the anti-inflammatory effect, suggesting that Nd doping plays a key anti-inflammatory role in macrophages (Fig. S3). The most significant immunomodulatory effects were observed in LNDMSN. Therefore, the low concentration of NDMSN was selected for immunofluorescence analysis. The immunofluorescence images confirmed the lower expression iNOS (lower fluorescence intensity) and the higher expression CD206 (higher fluorescence intensity) in NDMSN-treated M1 macrophages compared with the M1 macrophages, and the quantitative analysis confirmed significant reduction levels of iNOS and promotion levels of CD206 between the NDMSN-treated M1 macrophage group and the M1 macrophage group (Fig. 2B and Fig. S4). Quantitative flow cytometric analysis demonstrated that LPS stimulation markedly elevated the M1 macrophage population (CD86^+^ cells) to 38.9 %, compared to untreated controls. In contrast, LNDMSN treatment enhanced the proportion of M2 macrophages (CD206-positive) to 23.8 % while reducing the proportion of M1 macrophages to 13.6 % and 47.9 % of the cells treated with LNDMSN express both CD206 and CD86 (Fig. 2C). Additionally, ELISA analysis revealed that LNDMSN treatment significantly increased TGF-β and IL-10 secretion in M1 macrophage culture supernatants compared to untreated controls (Fig. S5). These results strongly confirm that NDMSN (25 μg/mL) significantly switched the macrophages from pro-inflammatory to anti-inflammatory.

### 3.3 Autophagy-mediated immunomodulatory response by NDMSN

**Fig. 3:**
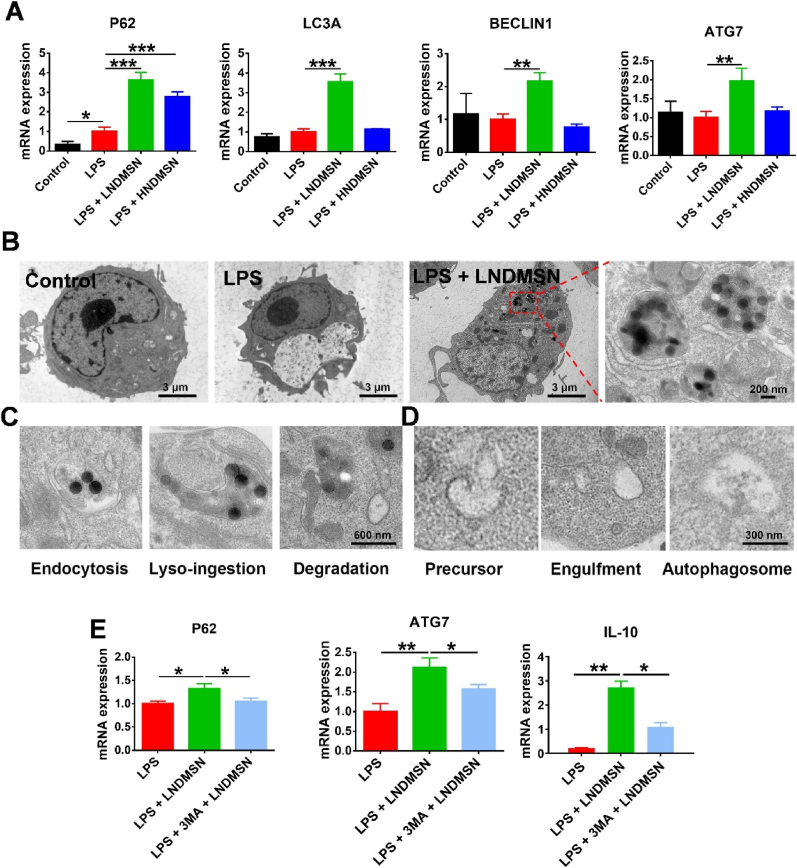
**Autophagy-mediated immunomodulatory response by NDMSN.** (A) RT-qPCR results of autophagy gene expressions (P62, LC3A, BECLIN1, and ATG7) in macrophages. (B) TEM images of macrophages treated with Control (PBS), LPS, and LPS + LNMSN for 24 h. (C) The uptake and destruction of NDMSN in M1 macrophages. (D) Formation of autophagosome in macrophages under NDMSN induction. (E) P62, ATG7, and IL-10 mRNA expression in macrophages. Data are expressed as mean ± standard deviation, n = 3. There is a substantial difference between the groups, with significance levels of ∗p < 0.05, ∗∗p < 0.01, and ∗∗∗p < 0.001.

The mechanism underlying the immunomodulation by NDMSN was studied by analyzing the cellular autophagic response. The quantitative RT-qPCR tests revealed that the LPS-treated group had higher levels of the autophagy marker P62 mRNA expression compared to the control group (Fig. 3A). Both low and high NDMSN concentrations significantly increased the autophagy gene P62 expression. At the same time, treating cells with LNDMSN also increased the expression of autophagy markers LC3, BECLIN1, and ATG7 compared to the group that was only treated with LPS (Fig. 3A). Western blot quantification results indicated that LNDMSN inhibits P62 and promotes secretion of autophagy markers LC3 and BECLIN1 protein expressions (Fig. S6). TEM was used to observe the intracellular distribution of the nanoparticles to study further the occurrence of autophagy induced by NDMSN on macrophages. NDMSN nanoparticles were internalized in LPS-treated macrophages (Fig. 3B), and at all stages of nanoparticle internalization, degradation, and autophagy formation by macrophages could be observed (Fig. 3C and D). These results exhibited that NDMSN has a robust autophagy-inducing ability. To study more about how NDMSN-induced autophagy and anti-inflammatory reactions work together, the enzyme 3 MA was used to stop macrophage autophagy. Blocking autophagy showed significant inhibitions of the NDMSN-induced mRNA levels of the autophagy markers P62 and ATG7 (Fig. 3E). ELISA results further confirmed that inhibition of autophagy reduced the NDMSN-induced expression of the M2 macrophage marker IL-10 (Fig. 3E). These results suggest that NDMSN causes an anti-inflammatory reaction in M1 macrophages via the activation of autophagy.

### 3.4 NDMSN-mediated macrophage immunomodulation promoted the angiogenic differentiation of HUVECs

**Fig. 4:**
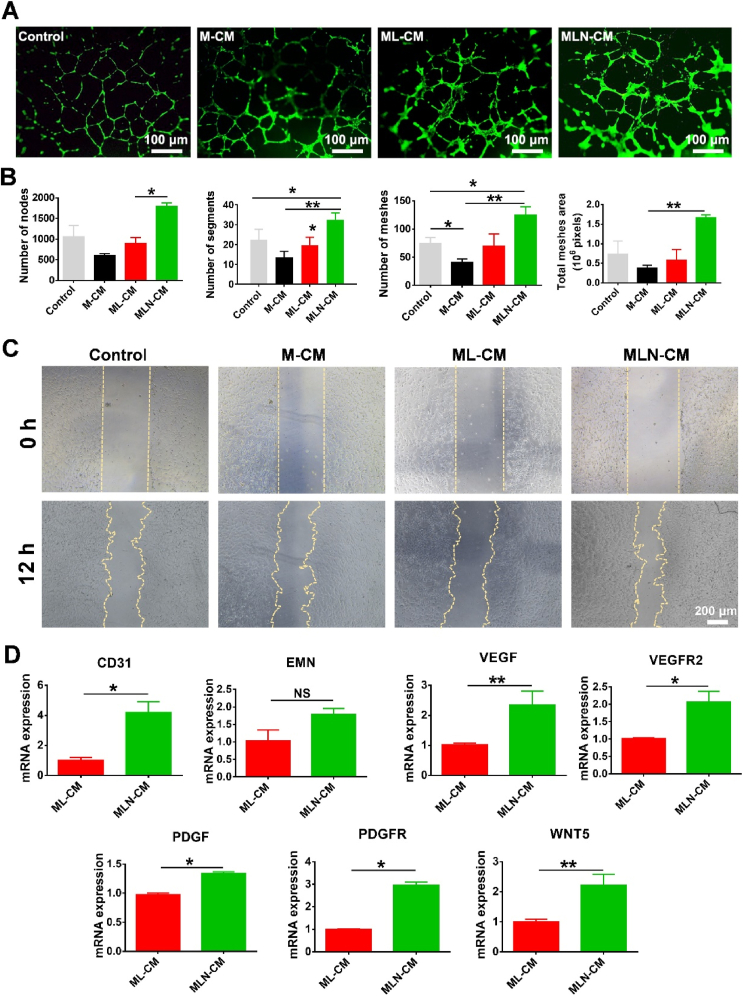
**NDMSN-treated macrophage-conditioned medium (CM) promotes angiogenic differentiation of HUVECs.** (A) Matrigel gel tube formation assay. (B) Statistical analysis of the number of nodes, segments, meshes, and total mesh area. (C) Scratch wound migration assay with HUVECs; (D) RT-qPCR analysis of CD31, EMN, VEGF, VEGF2, PDGF, PDGFR, and WNT5. Control: non-conditioned medium; M-CM: conditioned medium from macrophages treated with PBS for 24 h; ML-CM: conditioned medium from macrophages treated with LPS for 24 h; MLN-CM: conditioned medium from macrophages treated with LPS for 24 h, followed by an 8 h treatment with NDMSN. Data are mean ± SD, n = 3. Significant difference between the groups,∗p < 0.05, ∗∗p < 0.01, and ∗∗∗p < 0.001.

The in vitro Matrigel tube formation assay with HUVECs was performed to analyze the angiogenic efficacy of the conditioned media of three treatment groups: macrophages only (M-CM), macrophages induced by LPS (ML-CM), and LPS-induced macrophages treated with NDMSN (MLN-CM). The control condition was a non-conditioned medium. The images and statistical analyses showed that the conditioned medium obtained from LPS-treated or untreated macrophages did not inhibit or equal the HUVECs’ angiogenic differentiation compared with the control group (Fig. 4A and B). The M-CM decreased the number of nodes, segment number meshes, and total mesh area most substantially compared to the control condition. At the same time, the ML-CM group showed similar values for most of these parameters (Fig. 4B). However, the MLN-CM group exhibited significant enhancement of tube formation, an increased number of nodes compared with the control group, a higher number of segments and meshes compared with both the M-CM and ML-CM group, and an elevated total mesh area compared with the ML-CM group (Fig. 4B). The scratch assay demonstrated that conditioned medium derived from NDMSN-treated M1 macrophages (induced by LPS) significantly promoted the migration of HUVECs (Fig. 4C). With RT-qPCR, it was found that MLN-CM enhanced the angiogenic markers CD31, EMN, VEGF, VEGFR2, PDGF, PDGFR, and WNT5 compared with ML-CM (Fig. 4D). These results indicate that NDMSN can induce angiogenic differentiation of HUVECs by modulating the macrophage immune environment.

### 3.5 NDMSN-mediated macrophage immunomodulation promoted osteogenic differentiation of BMSCs

**Fig. 5:**
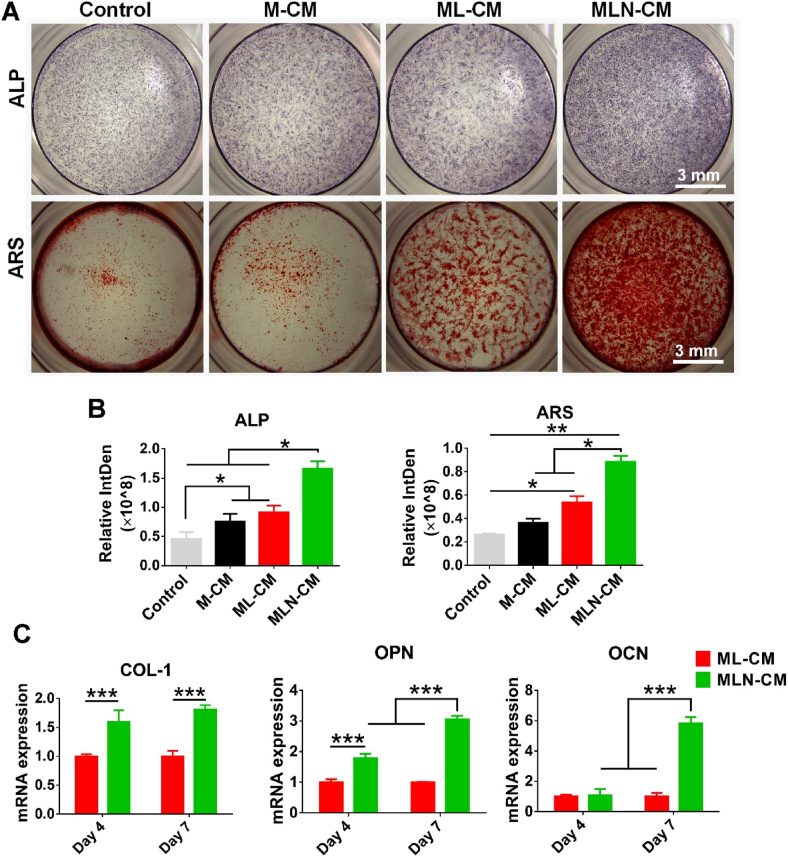
**NDMSN-treated macrophage-conditioned medium (CM) promotes osteogenic differentiation of BMSCs.** (A) Alkaline phosphatase (ALP) staining and alizarin red S (ARS) staining after 14 days of culture. (B) Quantification of ALP and ARS intensities. (C) mRNA expression patterns of osteogenic markers COL-1, OPN, and OCN in BMSCs. Data are mean ± SD, n = 3. Significant difference between the groups,∗p < 0.05, ∗∗p < 0.01, and ∗∗∗p < 0.001. Control: non-conditioned medium; M-CM: PBS-treated group; ML-CM: LPS-treated group; MLN-CM: LPS + NDMSN-treated group.

MLN-CM-treated BMSCs showed higher ALP production than M-CM, ML-CM, and control medium-treated BMSCs (Fig. 5A and B). ARS images showed higher mineralized matrix intensity in MLN-CM versus ML-CM cultured BMSCs, while BMSCs cultured in M-CM and Control medium showed only marginal alizarin red staining (Fig. 5A and B). Similarly, mRNA expression levels of osteogenic markers COL-1, OPN, and OCN were significantly upregulated in the MLN-CM group compared to the ML-CM group (Fig. 5C). The Western blot analysis results indicated that MLN-CM upregulates OPN expression compared with ML-CM group (Fig. S7). In addition, MLM-CM did not affect osteogenic differentiation of BMSCs (Fig. S8). These results suggest that NDMSN-induced macrophage immunomodulation enhanced osteogenic differentiation of BMSCs.

### 3.6 NDMSN inhibited the LPS-induced calvarial bone destruction

**Fig. 6:**
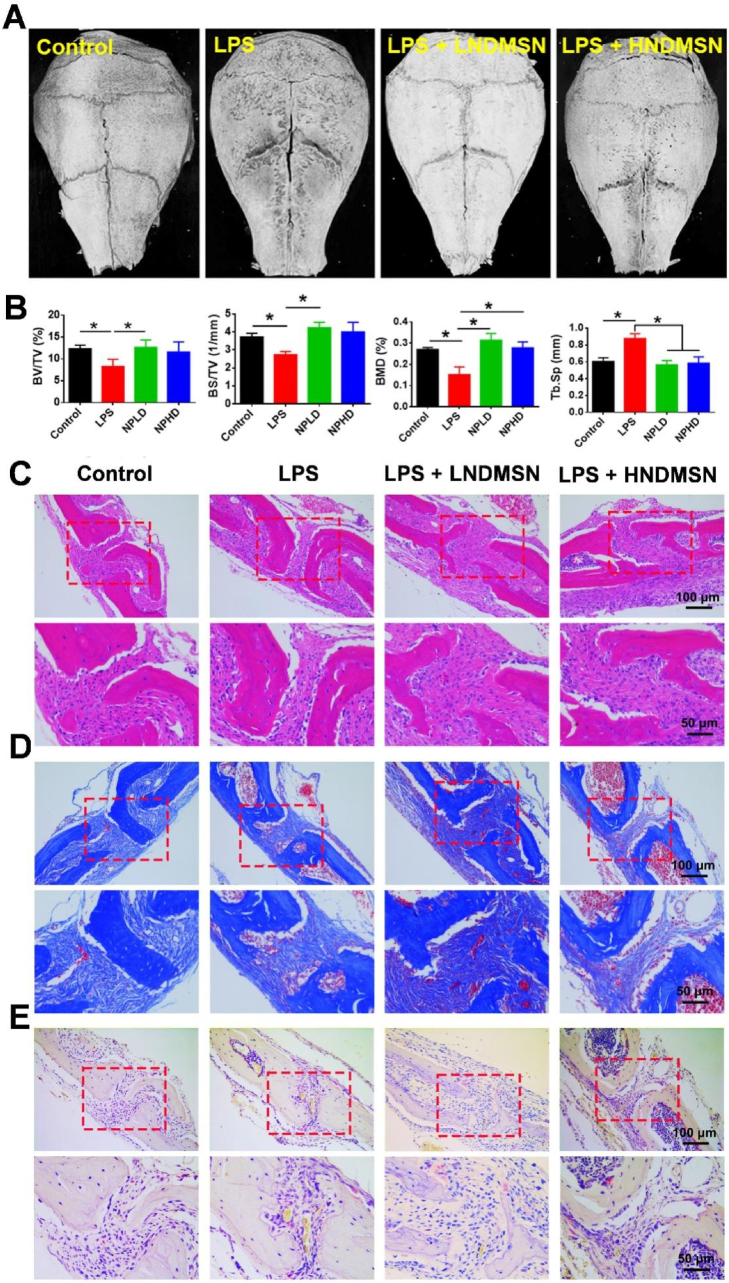
**NDMSN-mediated immunomodulation attenuates LPS-induced osteolysis in the mouse calvaria.** (A) Representative 3D reconstructed micro-CT images of mouse calvarial bone defect after 10 days of treatment. (B) Morphometric analysis of BV/TV, BS/TV, BMD, and Tb. Sp. (C) The H&E staining, (D) Masson trichrome staining, and (E) Tartrate-resistant acid phosphatase staining (TRAP). Data are mean ± SD, n = 6. Significant difference between the groups,∗p < 0.05.

LNDMSN (low dose NDMSN, 25 μg/kg body weight) or HNDMSN (high dose NDMSN, 100 μg/kg body weight) were injected in a mouse calvarial osteolysis model to test the effects of Nd-doped mesoporous silica nanoparticles. The micro-CT reconstruction images revealed that maximum bone erosion was observed on the calvarial bone surface in the LPS group, which displayed deep and extensive resorption pits (Fig. 6A and Fig. S9). In contrast, mouse calvaria treated with LNDMSN showed a significant reduction in loss of calcified bone tissue, ultimately ameliorating LPS‐induced bone destruction (Fig. 6A). Analysis of bone morphology revealed that co-administration of LPS and LNDMSN significantly improved structural and mineral parameters in mouse calvaria compared to LPS monotherapy, including increased bone volume (BV/TV), Bone surface volume (BS/TV), and Bone mineral density (BMD) (Fig. 6B). In agreement with these aspects, trabecular separation was markedly increased in the LPS-only group, which was reduced compared to control levels in LNDMSN-treated animals (Fig. 6B). The HNDMSN group exhibited similar patterns to those of the LNDMSN group. (Fig. 6B). Histological evaluation showed that calvaria treated with and without NDMSN displayed a decrease in fibrous tissues and a smaller number of osteoblasts in the LPS group compared to the control and LPS + LNDMSN group (Fig. 6C). The Masson trichrome staining further confirmed that the abundance of fibrous tissue consisting of collagen fibrils was denser in control and LNDMSN-treated after LPS injection compared to the LPS group (Fig. 6D). The TRAP staining and quantification showed a higher number of osteoclasts (red color TRAP-positive cells) in LPS-treated mouse calvaria, whereas LNDMSN and HNDMSN treatment substantially reduced osteoclast number (Fig. 6E and Fig. S10).

**Fig. 7:**
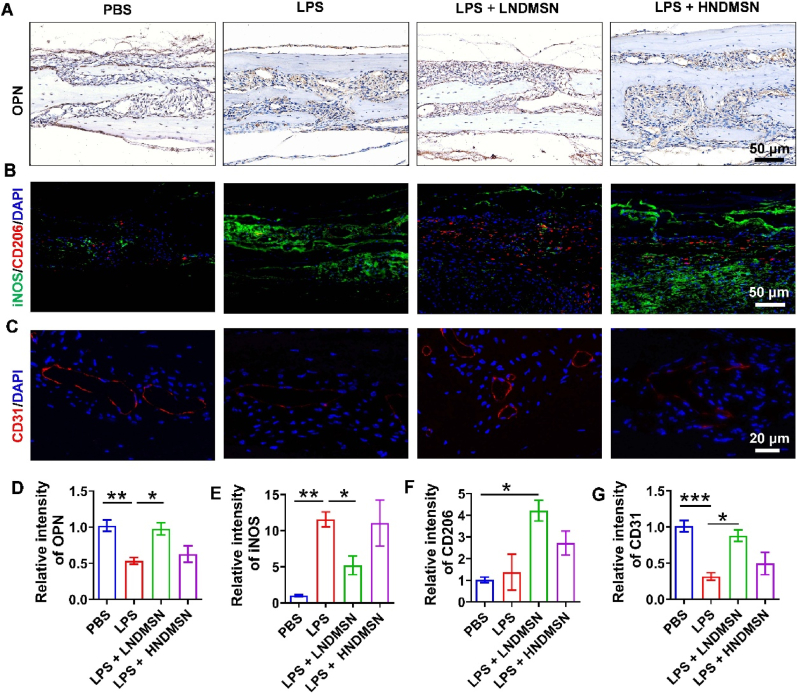
**NDMSN exerts anti-inflammatory and vessel regeneration effects *in vivo*.** (A) OPN immunochemistry staining; (B) iNOS (green) and CD206 (red) immunofluorescence co-localization staining and (C) CD31 (red) immunofluorescence staining; Quantitative analysis of (D) OPN, (E) iNOS, (F) CD206, and (G) CD31. Data are mean ± SD or SEM, n = 3. Significant difference between the groups, ∗*p* < 0.05, ∗∗*p* < 0.01, and ∗∗∗*p* < 0.001.

The OPN immunochemistry staining and quantification results showed that LPS inhibits OPN expression and LNDMSN reverses this effect (Fig. 7A and D). The iNOS and CD206 immunofluorescence staining showed a higher iNOS expression and a lower CD206 expression in LPS-group, whereas LNDMSN treatment reverse this effect (Fig. 7B, E, and 7F). The CD31 immunofluorescence staining showed that LNDMSN can significantly reverse LPS-inhibited CD31 expression (Fig. 7C and G). These results confirm that NDMSN suppressed the LPS-induced bone loss by suppressing osteoclast activity and promoting new bone formation and accumulation of fibrous connective tissues by collagen expression. The *in vivo* findings support the potential utility of NDMSN in addressing bone loss caused by inflammation.

### 3.7 NDMSN promoted zebrafish skull defect repair

**Fig. 8:**
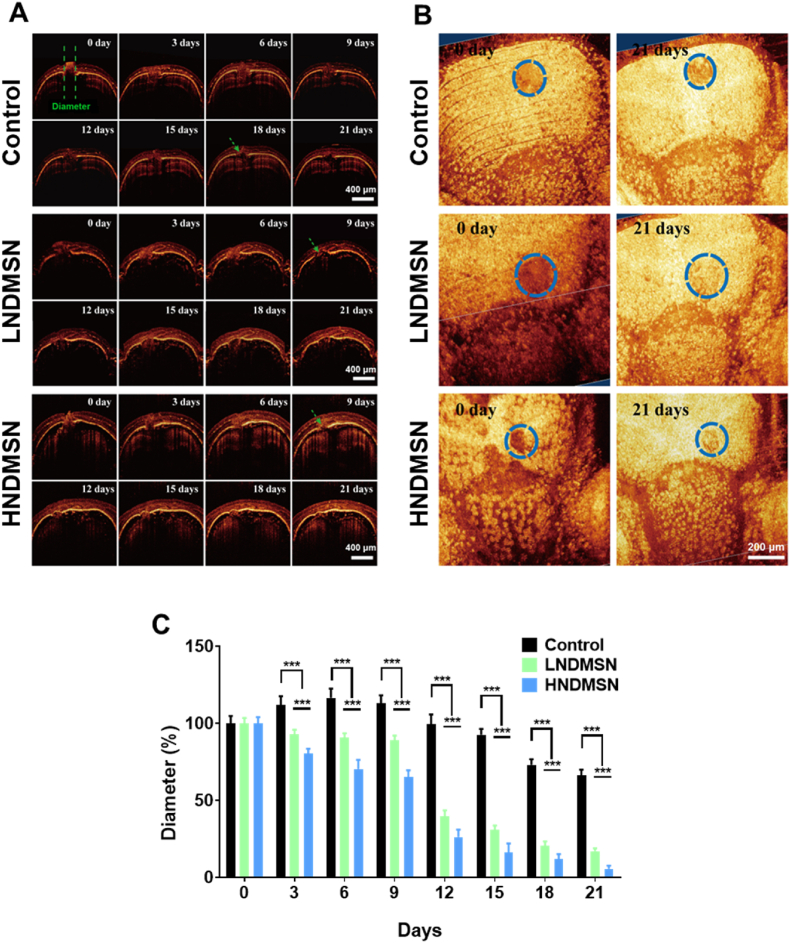
**NDMSN treatment promotes bone regeneration in the zebrafish skull defect model.** (A) Optical coherence tomography (OCT) cross-sectional images. The green arrow indicates the regeneration of the bone tissue. (B) 3D images of zebrafish skull defects. The dashed circles demarcate the extent of the injured region. (C) Skull defect diameter measured from OCT images of zebrafish. Data are mean ± SD, n = 10. Significant difference between the groups, ∗∗∗p < 0.001.

OCT images show the repair process of zebrafish bone defect (green vertical parallel line) after treatment with water (control), LNDMSN, or HNDMSN, respectively. Bone defects in the control group did not heal for a long time after injury, and new bone formation could only be observed at 18 days follow-up (FU; green arrow), while in the LNDMSN and HNDMSN groups, new bone formation was already observed at 9 days FU (Fig. 8A and Fig. S11). In addition, after 21 days of treatment, it was observed that most of the bone defects in the NDMSN treatment groups had recovered, while in the control group, more significant bone defects were still observed (Fig. 8A). Three-dimensional OCT images further proved that at 21 days FU, bone defects in the LNDMSN treatment group were basically healed, and those in the HNDMSN were partially healed, while in the control group, more significant bone defects were still present (Fig. 8B). Further quantitative analysis of the bone defect diameter in the OCT images of each treatment group showed that in the control group, the bone defect length increased first, then gradually decreased after 9 days, and the final bone defect length was the largest on day 9 (Fig. 8C). However, the LNDMSN and HDMSN groups had significant bone repair effects from day 3, while the diameter of bone defect at day 21 was less than 10 % of the initial diameter. Between the high and low-dose groups, the low-dose group showed the best therapeutic effects (Fig. 8C). These results indicate that NDMSN could promote bone regeneration of zebrafish skull defects.

## 4 Discussion

**Fig. 9:**
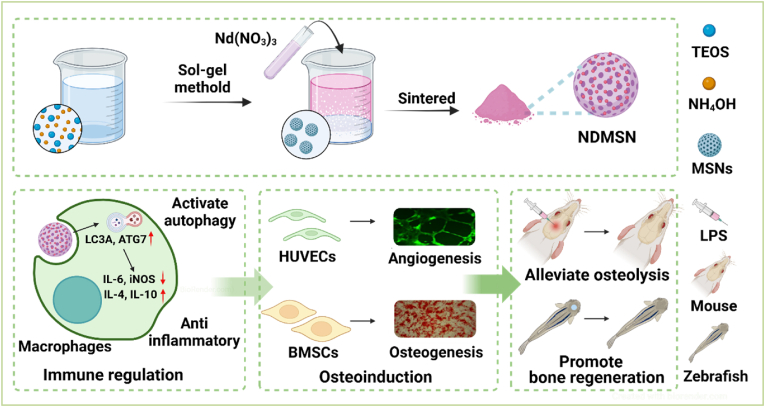
Schematic illustration of the synthesis of NDMSNs and thir effects on immune regulation-mediated angiogenesis and osteoinduction to alleviate inflammation-induced osteolysis and promote bone regeneration.

In this study, we investigated the bone immunomodulatory potential of Nd-based nanomaterials by developing Nd-doped mesoporous silica nanoparticles (NDMSN) with low cytotoxicity. Our findings demonstrate that NDMSN, at a non-toxic concentration (25 μg/mL), effectively modulates innate immune responses by polarizing M1 macrophages toward an intermediate anti-inflammatory M2 phenotype. This shift was characterized by suppressing pro-inflammatory mediators (IL-6, IL-1β, and iNOS) and the induction of anti-inflammatory markers (IL-4, IL-10, and CD206), a process mediated by autophagy. Furthermore, NDMSN-driven immunomodulation enhanced the angiogenic and osteogenic differentiation of precursor cells. *In vivo*, NDMSN mitigated LPS-induced osteolysis in a mouse calvarial model and promoted bone repair in a zebrafish defect model. Collectively, these results establish NDMSN as a promising nanomaterial for preventing bone loss through autophagy-dependent macrophage polarization, which fosters a regenerative microenvironment by coupling osteogenesis and angiogenesis (Fig. 9).

While previous studies have primarily examined the direct influence of Nd-based nanomaterials on angiogenesis [38] and osteogenic differentiation of precursor cells [20,21], our work explores their immunomodulatory role in macrophage-mediated bone regeneration. Upon entering the body, macrophages frequently phagocytose nanomaterials, limiting their accumulation at the target site [39]. Thus, a significant challenge in regenerative medicine lies in developing nanomaterials capable of evading immune clearance or actively modulating immune responses [16]. Given the critical role of immunoregulation in bone homeostasis, resorption, and repair [11], nanomaterial design strategies have shifted from passive immune evasion to active immunomodulation. Specifically, promoting macrophage polarization from pro-inflammatory M1 (which hinders regeneration) toward low-inflammatory or anti-inflammatory M2 phenotypes (which support regeneration) has emerged as a promising approach [33]. In our study, LNDMSN significantly promoted macrophage polarization toward the M2 phenotype. Notably, 47.9 % of NDMSN-treated macrophages co-expressed both CD206 and CD86 markers, indicating a mixed M1/M2 phenotype. This finding highlights the plasticity of macrophages, which can exhibit transitional states in response to varying stimuli [40]. Rather than strictly conforming to M1 or M2 classifications, macrophages may dynamically balance pro-inflammatory and reparative functions. Such a mixed polarization state could enhance their adaptability to shifting immune signals within the bone regeneration microenvironment, facilitating both tissue repair and immune modulation. The capacity of NDMSN to modulate macrophage immune responses may be pivotal to its therapeutic efficacy in regenerative medicine.

However, the clinical translation of Nd nanoparticles has been hindered by their cytotoxicity [5]. Previous studies indicate that Nd oxide nanoparticles suppress 16HBE cell proliferation at concentrations as low as 20 μg/mL [24,25] and exhibit toxicity in rat alveolar macrophages above 6.5 μg/mL [26]. To overcome this limitation, we employed an element-doping strategy, incorporating Nd into mesoporous silica nanoparticles (NDMSN) to mitigate its adverse effects. This approach aligns with recent advances in doping bioactive ions such as Cu, Sr, Zn, Ce, and Ru into MSNs, which have expanded their therapeutic potential [27,[41], [42], [43], [44], [45]]. The inherent biocompatibility of MSNs and the stable covalent bonding within their silicon-oxygen framework enable controlled ion release [46]. The chosen concentrations of 25 μg/mL (low) and 100 μg/mL (high) were determined based on preliminary cytotoxicity assessments. While Nd doping may marginally enhance the cytotoxic potential of MSNs, our results indicated that NDMSN did not cause significant cytotoxicity within 24 h, the timeframe used for all macrophage polarization and autophagy-related experiments. Regarding the *in vivo* dosage, 100 μg/kg NDMSN was selected based on prior studies confirming its biological efficacy and short-term biocompatibility. For instance, mesoporous silica nanoparticles (MSNs) have exhibited good biocompatibility *in vivo* at a dose of 100 μg/kg [47]. Furthermore, other Nd-doped biomaterials, including hydrogels and metallic implants, have also demonstrated favorable biocompatibility [48,49]. While NDMSN demonstrated dose- and time-dependent cytotoxicity in vitro. These results underscore the potential of element-doped silica nanoparticles as a viable platform for harnessing the therapeutic benefits of rare earth elements while minimizing their cytotoxicity.

Successful bone defect reconstruction relies on three key processes: osteogenic differentiation, angiogenesis [46], and suppression of osteoclast activity [50,51]. Our study demonstrates that conditioned medium from NDMSN-polarized M1 macrophages significantly enhanced angiogenic differentiation and upregulated angiogenic gene expression in HUVECs. This aligns with previous reports showing that Nd nanoparticles directly stimulate angiogenesis by increasing pro-angiogenic factors such as VE-cadherin, HIF-1α, VEGF, and VEGFR-2 in HUVECs [38]. These observations suggest that NDMSN may exert both direct and indirect pro-angiogenic effects, though further mechanistic studies are warranted. The conditioned medium from NDMSN-treated macrophages also promoted osteogenic differentiation and upregulated osteogenic gene expression in BMSCs. Supporting these findings, recent studies have shown that hydrogels incorporating Nd and manganese ions significantly enhance hBMSC viability and osteogenic differentiation compared to undoped hydrogels [20], reinforcing the therapeutic potential of Nd-based biomaterials in bone regeneration. Although M2 polarization is widely linked to regenerative processes, evidence indicates that M2 activation alone may not fully explain the observed pro-angiogenic and pro-osteogenic effects. For instance, while IL-4 induced M2 polarization has been shown to enhance neovascularization and bone regeneration *in vivo* [52], the magnitude of this effect depends on additional microenvironmental signals. In our study, NDMSN-conditioned medium exhibited increased levels of IL-10 and TGF-β (Fig. S5), two key cytokines known to stimulate endothelial STAT3 signaling and angiogenesis [53,54]. Furthermore, this conditioned medium significantly improved tube formation in HUVECs and upregulated osteogenic genes in BMSCs, underscoring the critical role of macrophage-derived soluble factors in shaping the regenerative microenvironment beyond a simple M1/M2 dichotomy. These findings suggest that NDMSN reprograms macrophage function to secrete pro-regenerative cytokines, which collectively regulate endothelial and mesenchymal cell behavior to promote bone tissue regeneration.

Beyond facilitating bone repair and regeneration, effective therapeutic strategies must also prevent excessive osteoclast-mediated bone resorption, which is frequently exacerbated under inflammatory conditions. In LPS-induced osteolysis models, NDMSN treatment markedly reduced TRAP-positive osteoclast formation, demonstrating its protective effect against pathological bone loss. Mechanistically, NDMSN attenuated the pro-inflammatory response of M1 macrophages and enhanced the secretion of anti-inflammatory cytokines (IL-4 and IL-10), both of which are established inhibitors of osteoclastogenesis [55,56]. IL-4 suppresses osteoclast differentiation via type I receptor signaling [56], while IL-10 modulates the MEG3/IRF8 pathway to inhibit osteoclast activity and bone resorption [55]. Furthermore, silicon ions (Si) released from the MSN matrix may contribute to osteoclast inhibition, as evidenced by similar effects in bioglass systems [57]. Thus, NDMSN likely exerts its anti-osteoclastogenic effects through a synergistic mechanism: immunomodulation of macrophage polarization coupled with Si ion release. Our results and emerging literature indicate that rare earth-doped biomaterials enhance bone regeneration through multifaceted actions, i.e., directly influencing precursor cell differentiation while indirectly creating a pro-regenerative microenvironment.

Our zebrafish cranial defect model revealed NDMSN's significant regenerative capacity. Control animals showed progressive defect expansion, with initial defect dimensions only restored after 15 days - a delay we attribute to persistent inflammatory responses that may maintain a degenerative state. Notably, recovery initiation occurred only after day 9 in controls. In striking contrast, NDMSN-treated specimens demonstrated immediate regenerative progression, achieving near-complete defect resolution within 21 days. This accelerated healing trajectory strongly supports NDMSN's immunomodulatory mechanism in bone regeneration, where early inflammation control appears crucial for initiating and sustaining the repair process.

Our study reveals that NDMSN activates autophagy, as demonstrated by increased expression of LC3A, Beclin1, and ATG7. Intriguingly, while p62 mRNA levels rose, its protein levels declined, a paradoxical observation given that p62 is a canonical autophagy substrate typically degraded during autophagic flux. This discrepancy suggests that NDMSN enhances p62 protein degradation while triggering compensatory transcriptional upregulation. Notably, the autophagy inhibitor 3-MA abolished NDMSN-induced p62 mRNA elevation, confirming that this transcriptional response depends on active autophagy. This aligns with prior studies reporting that prolonged autophagy can induce p62 mRNA upregulation alongside sustained protein degradation [58,59]. Collectively, these results indicate that NDMSN enhances functional autophagic flux rather than impairing degradation, a mechanism that may contribute to its immunomodulatory effects in macrophages.

This study demonstrates that NDMSN effectively promote bone regeneration through immunomodulatory mechanisms, where, within a safe concentration range, they regulate macrophage polarization toward an anti-inflammatory phenotype via autophagy activation, thereby inhibiting inflammation-induced bone destruction while simultaneously enhancing immune-mediated angiogenic and osteogenic differentiation. Our findings demonstrate that the developed nanomaterials show strong potential for clinical applications, particularly in treating critical-sized bone defects requiring intervention. These materials may also prove valuable for inflammatory bone disorders like periodontitis and osteomyelitis, where combined immune modulation and osteogenic enhancement are crucial therapeutic targets. Due to their dual bioactivity and immunomodulatory capacity, these nanomaterials could be effectively incorporated into scaffold matrices or injectable delivery systems, enabling targeted therapy with reduced risk of systemic effects. While these results underscore the therapeutic promise of NDMSN, further investigation is required to evaluate long-term exposure effects, biodistribution patterns, and systemic safety profiles of Nd-doped nanoparticles before clinical translation. Moreover, the specific contribution of local macrophage modulation during *in vivo* bone defect repair remains to be fully elucidated, necessitating further investigation using macrophage-depleted animal models to comprehensively evaluate the bone healing capacity of NDMSN in the absence of macrophage-mediated immune responses.

## 5 Conclusion

Our study presents the development and comprehensive evaluation of NDMSN as novel osteoimmunomodulatory biomaterials. Through systematic investigation, we established that NDMSN exhibit remarkable biocompatibility while effectively modulating macrophage polarization through autophagy activation, converting pro-inflammatory macrophages toward an anti-inflammatory phenotype. The therapeutic potential of NDMSN was further evidenced by their ability to promote both angiogenesis and osteogenesis, as demonstrated by enhanced differentiation of precursor cells exposed to conditioned medium from NDMSN-treated macrophages. In translational animal models, NDMSN showed significant efficacy in bone regeneration, successfully reversing LPS-induced osteolysis in mouse calvaria, as evidenced by restored bone mineral density and volume, and accelerating cranial defect repair in zebrafish models. These multifaceted findings position NDMSN as a promising platform for bone tissue engineering, offering simultaneous immunomodulation and osteogenic stimulation through a single biomaterial approach. The demonstrated capacity to coordinate both immune microenvironment regulation and direct tissue regeneration underscores the potential of NDMSN to address critical challenges in bone defect repair and inflammatory bone disease treatment.

## CRediT authorship contribution statement

**Qing Zhang:** Writing – original draft, Methodology, Funding acquisition, Formal analysis, Data curation. **Duraipandy Natarajan:** Writing – original draft, Methodology, Investigation. **Weijian Gao:** Methodology. **Haokun He:** Methodology. **Shuguang Cheng:** Methodology. **Yin Xiao:** Writing – review & editing, Conceptualization. **Marco N. Helder:** Writing – review & editing, Conceptualization. **Sujuan Zeng:** Writing – review & editing. **Richard T. Jaspers:** Writing – review & editing, Conceptualization. **Janak Lal Pathak:** Writing – original draft, Funding acquisition, Conceptualization.

## Funding

This study was funded by the 10.13039/501100001809National Natural Science Foundation of China (32301129 and 82150410451), the 10.13039/501100010256Guangzhou Science and Technology Plan Project (2023B03J1240), and the Science and Technology Projects in Guangzhou (202201020396).

## Declaration of competing interest

The authors declare that they have no known competing financial interests or personal relationships that could have appeared to influence the work reported in this paper.

## Data Availability

Data will be made available on request.
