# Supplementary material for: Neodymium-doped mesoporous silica nanoparticles promote bone regeneration via autophagy-mediated macrophage immunomodulation
Source: Mater Today Bio. 2025 Aug 12;34:102198. doi: 10.1016/j.mtbio.2025.102198 (PMC12390955; doi:10.1016/j.mtbio.2025.102198)
Supplement: Multimedia component 1 [file mmc1.docx]

**Supplementary Information**

**Neodymium-Doped Mesoporous Silica Nanoparticles Promote Bone Regeneration via Autophagy-Mediated Macrophage Immunomodulation**

Qing Zhang^1,2, †^, Duraipandy Natarajan^1, †^, Weijian Gao^3^, Haokun He^1^, Shuguang Cheng^1^, Yin Xiao ^1, 4^, Marco N Helder ^5^, Sujuan Zeng^1^*, Richard T Jaspers ^1, 2^*, Janak Lal Pathak^1^*

^1^School and Hospital of Stomatology, Guangdong Engineering Research Center of Oral Restoration and Reconstruction, Guangzhou Key Laboratory of Basic and Applied Research of Oral Regenerative Medicine, Guangzhou Medical University, Guangzhou, 510182, China

^2^Laboratory for Myology, Department of Human Movement Sciences, Faculty of Behavioural and Movement Sciences, Vrije Universiteit Amsterdam, Amsterdam Movement Sciences, 1081 BT Amsterdam, The Netherlands

^3^School of Biomedical Engineering, Guangzhou Medical University, Guangzhou 511436, Guangdong, China

^4^School of Medicine and Dentistry & Institute for Biomedicine and Glycomics, Griffith University, Gold Coast, QLD 4222, Australia

^5^Department of Oral and Maxillofacial Surgery/Oral Pathology, Amsterdam University Medical Centers and Academic Centre for Dentistry Amsterdam (ACTA), Vrije Universiteit Amsterdam, Amsterdam Movement Sciences, 1081 HV Amsterdam, The Netherlands

***Corresponding authors**:

[j.pathak@gzhmu.edu.cn](mailto:j.pathak@gzhmu.edu.cn); r.t.jaspers@vu.nl; zengsujuan78@foxmail.com

^†^These authors contributed equally to this work

**Figure S1.** Neodymium (Nd) ions release rate of NDMSN in DMEM buffer solution.


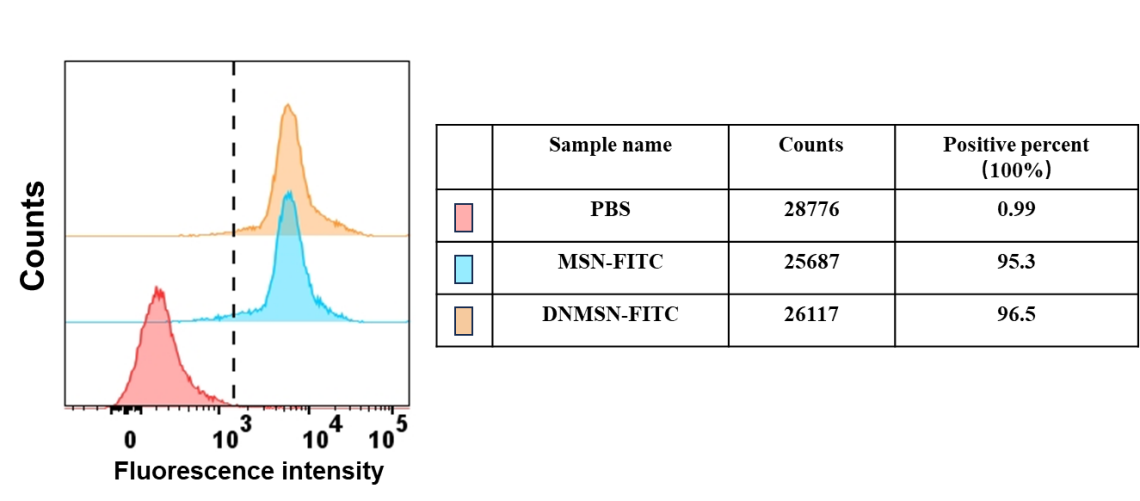


**Figure S2.** Flow cytometry to detected macrophages phagocytosed MSN and NDMSN.


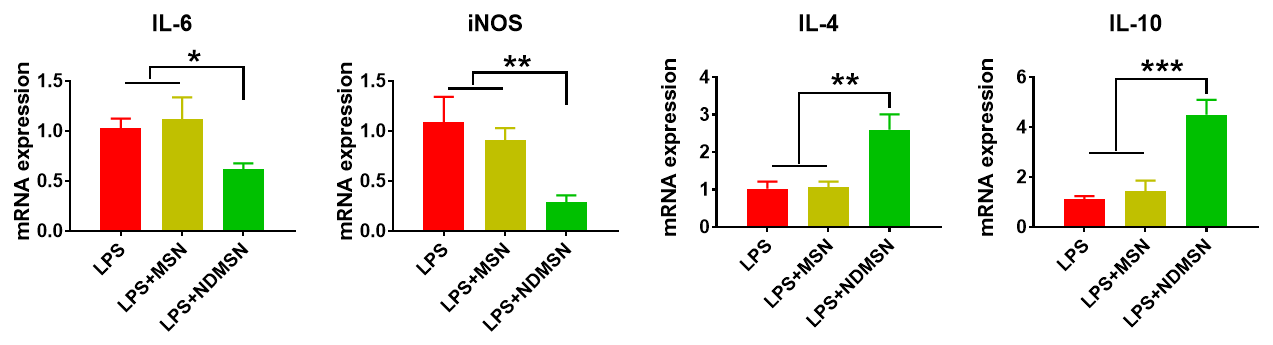


**Figure S3. MSN did not affect the macrophage polarization.** RT-qPCR results of proinflammatory markers (IL-6 and iNOS), and anti-inflammatory markers (IL-4 and IL-10). Data are mean ± SD, n = 3. Significant difference between the groups, *p < 0.05, **p < 0.01, and ***p < 0.001.


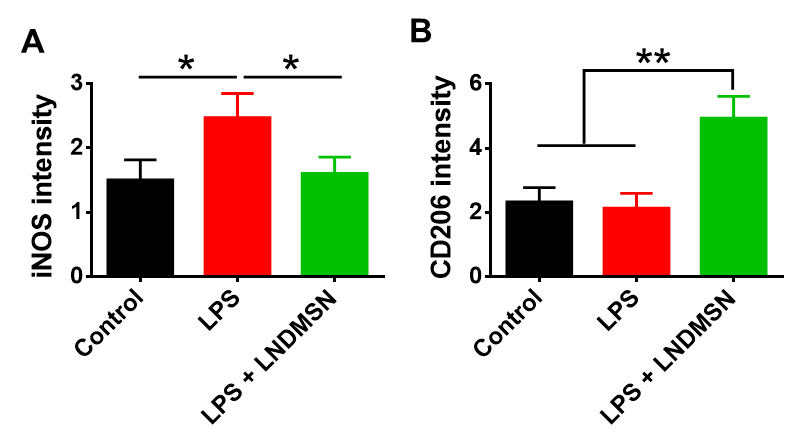


**Figure S4.** The quantitative statistical of iNOS (A) and CD206 (B) fluorescence intensity. Data are mean ± SD, n = 3. Significant difference between the groups, *p < 0.05 and **p < 0.01.


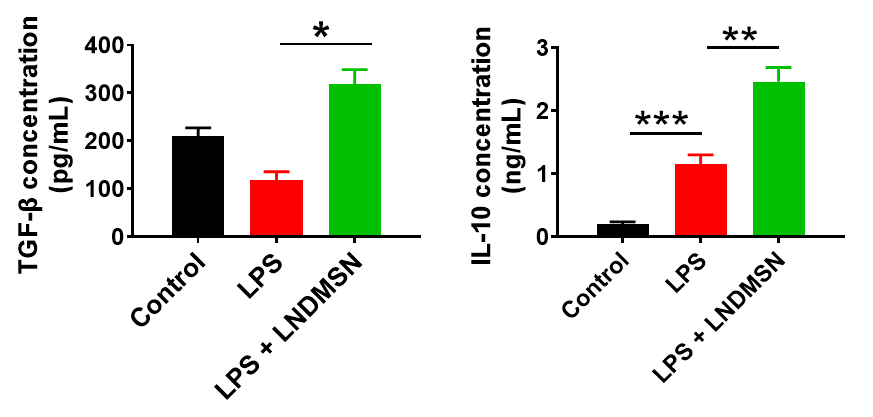


**Figure S5.** The concentration of TGF-β and IL-10 in the culture supernatant of macrophages cultured with Control, LPS, and LPS + LNDMSN for 24 h, respectively. D Data are mean ± SD, n = 3. Significant difference between the groups, *p < 0.05, **p < 0.01, and ***p < 0.001.


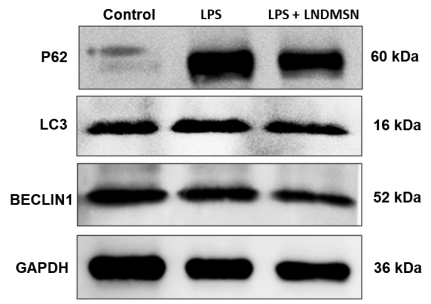

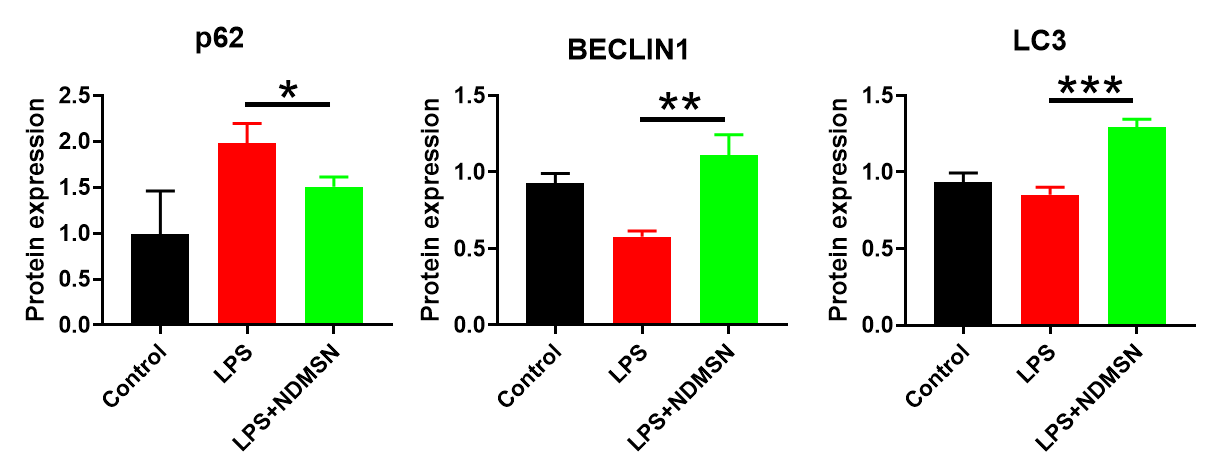


**Figure S6.** Western blot images and statistical analysis of protein level expression of P62, LC3, and BECLIN1. Data are mean ± SD, n = 3. Significant difference between the groups, *p < 0.05, **p < 0.01, and ***p < 0.001.


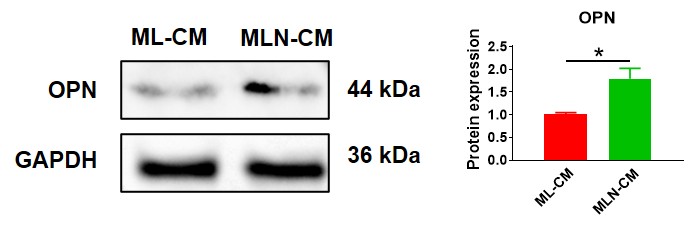


**Figure S7.** Western blot images and statistical analysis of protein level expression of OPN. Data are mean ± SD, n = 3. Significant difference between the groups, *p < 0.05. ML-CM: LPS-treated group; MLN-CM: LPS+NDMSN-treated group.


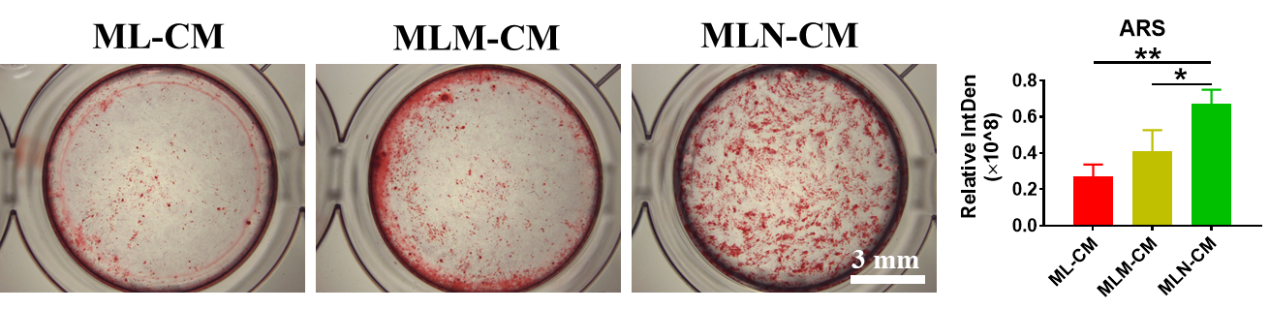


**Figure S8.**  ARS staining and statistic results of BMSCs treated with different osteogenic differentiation conditioned medium: ML-CM, MLM-CM, and MLN-CM respectively. Data are mean ± SD, n = 3. Significant difference between the groups, *p < 0.05 and **p < 0.01. ML-CM: LPS-treated group; MLM-CM: LPS+MSN-CM; MLN-CM: LPS+NDMSN-treated group.


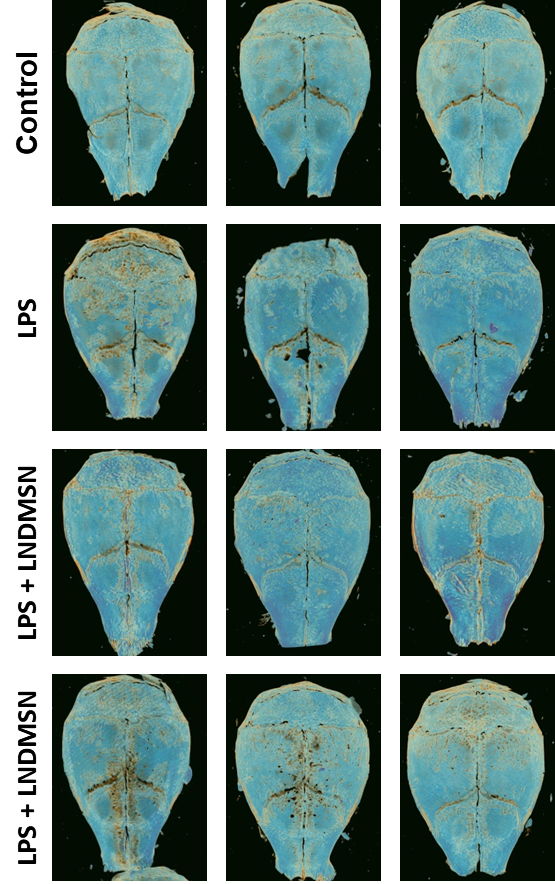


**Figure S9.** Representative 3D reconstructed µCT images of mouse calvarial bone defect after 10 days of treatment with control (PBS), LPS (10 mg/kg mouse), LPS + LNDMSN (25 µg/mL), and LPS + HNDMSN (100 µg/mL).

**Figure S10.** Osteoclast cells were quantified, Significant difference between the groups, *p < 0.05. Data are mean ± SD, n = 6. Significant difference between the groups,*p < 0.05.


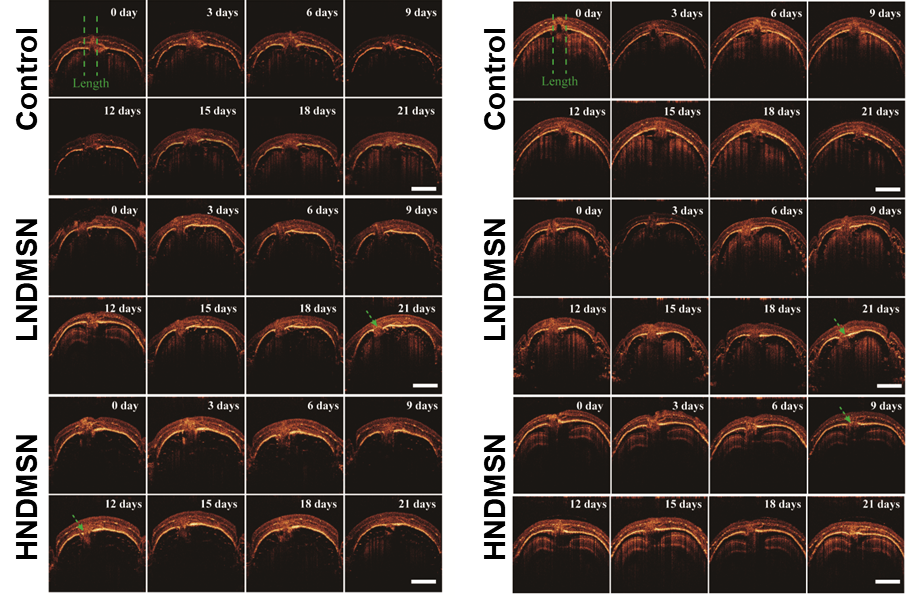


**Figure S11.** (A) Optical coherence tomography (OCT) cross-sectional images showed that NDMSN treatment promotes bone regeneration in the zebrafish skull defect model.
